# Relationship between social cognition and premorbid adjustment in psychosis: a systematic review

**DOI:** 10.1038/s41537-023-00428-y

**Published:** 2024-03-15

**Authors:** P. Punsoda-Puche, A. Barajas, M. Mamano-Grande, A. Jiménez-Lafuente, S. Ochoa

**Affiliations:** 1 Parc Sanitari Sant Joan de Déu, C/ del Dr. Antoni Pujadas, 42, 08830 Sant Boi de Llobregat, Barcelona, Spain; 2 Etiopatogènia i tractament dels trastorns mentals greus (MERITT), Institut de Recerca Sant Joan de Déu, C/ del Dr. Antoni Pujadas, 42, 08830 Sant Boi de Llobregat, Barcelona, Spain; 3 Department of Clinical and Health Psychology-Serra Húnter Programme, Universitat Autònoma de Barcelona, 08193 Cerdanyola del Vallès, Barcelona, Spain; 4 Department of Research, Centre d’Higiene Mental Les Corts, C/ de Numància, 107, 08029 Barcelona, Spain; 5 Investigación Biomédica en Red de Salud Mental (CIBERSAM), Barcelona, Spain

**Keywords:** Psychosis, Schizophrenia

## Abstract

This systematic review provides a comprehensive overview of the association between premorbid adjustment and social cognition in people with psychotic spectrum disorder. Obtaining evidence of this association will facilitate early detection and intervention before the onset of psychosis. Literature searches were conducted in Scopus, PubMed and PsycINFO. Studies were eligible if they included patients with a psychotic disorder or at a high-risk state; social cognition and premorbid adjustment were measured; and the relationship between premorbid adjustment and social cognition was analysed. The authors independently extracted data from all included articles, and discrepancies were resolved through discussion. Literature searches were conducted in Scopus, PubMed and PsycINFO. Studies were eligible if they included patients with a psychotic disorder or at a high-risk state; social cognition and premorbid adjustment were measured; and the relationship between premorbid adjustment and social cognition was analysed. The authors independently extracted data from all included articles, and discrepancies were resolved through discussion. Of 229 studies identified, 23 met the inclusion criteria. Different methods of assessment were used to measure premorbid adjustment, such as the Premorbid Adjustment Scale or premorbid IQ, among others. Social cognition was assessed as a global measure or by domains using different instruments. A total of 16 articles found a relationship between social cognition (or its domains) and premorbid adjustment: general social cognition (*n* = 3); Theory of Mind (*n*  = 12); Emotional Recognition and Social Knowledge (*n*  = 1). This review shows evidence of a significant relationship between social cognition and premorbid adjustment, specifically between Theory of Mind and premorbid adjustment. Social cognition deficits may already appear in phases prior to the onset of psychosis, so an early individualized intervention with stimulating experiences in people with poor premorbid adjustment can be relevant for prevention. We recommend some future directions, such as carrying out longitudinal studies with people at high-risk of psychosis, a meta-analysis study, broadening the concept of premorbid adjustment, and a consensual assessment of social cognition and premorbid adjustment variables. PROSPERO registration number: CRD42022333886.

## Introduction

Early detection and intervention before the onset of psychosis can have a positive impact on overall outcomes, symptoms and global functioning^1^. It is crucial to understand how the clinical debut of patients with psychosis occurs to study the relationship between present difficulties and difficulties in the past. Evidence of this link can provide adequate intervention and preventive strategies for psychosis.

Reduced cognitive function at an early age and academic underperformance are studied risk factors for developing schizophrenia^2^. A poorer premorbid adjustment has been described as a risk factor of poor outcome in early-onset psychosis^3^.

The first definitions of premorbid adjustment in schizophrenia contemplated the characteristics of a person, especially interpersonal relations and occupational functioning before the onset of schizophrenia^4^. Recent approaches consider that premorbid adjustment integrates psychosocial functioning in educational, occupational, social, and interpersonal relation areas^5,6^, considering premorbid adjustment a multidimensional factor. Dewangan et al.^7^ described premorbid adjustment as a combination of peer and social relationships, school adaptation, job functioning and opposite-sex relationship before the development of schizophrenia. Scientific evidence supports the existence of at least two domains of premorbid adjustment: academic and social, each of which has different correlates. For example, academic functioning is associated with neurocognitive functioning^8–10^. In this review, instead of academic premorbid adjustment, we considered a broader term: cognitive premorbid adjustment, which includes subjective information (academic premorbid adjustment), measured by a scale, and objective information (premorbid IQ), measured by a cognitive test.

Neurocognition encompasses all the basic processes that allow a person to learn about, understand, and know the world they live in^11^. Research in schizophrenia has identified eight separate neurocognitive domains, one of which is social cognition^12^. According to Green et al.^13^, social cognition has been defined as the mental operations underlying social interactions, such as perceiving, interpreting, and generating responses to the intentions and behaviours of others. There is consensus on four core domains of social cognition: emotion processing, social perception, Theory of Mind (mental state attribution) and attributional style/bias^14^. Emotion processing is defined as perceiving and using emotions. Social perception/knowledge refers to decoding and interpreting social cues in others. Theory of Mind is the ability to represent the mental states of others, including the inference of intentions and beliefs. Attributional style describes the way in which individuals explain the causes or make sense of social events or interactions.

There are no previous systematic reviews that study the relationship between social cognition and premorbid adjustment in people with psychosis. It has been described that social cognition is associated with social functional outcome^15^, but there is very scarce literature studying the relationship between social cognition and premorbid adjustment.

The aim of this review is to assess the relationship between social cognition after the onset of psychosis and premorbid adjustment in people with psychotic spectrum disorder. The specific aim of the study is to systematically review the relationship between social and academic premorbid adjustment and social cognition, and its domains in people with psychotic spectrum disorder. The finality of studying the link of social cognition and premorbid adjustment will give evidence to study the predictive capacity of the course of psychosis, help clinicians achieve early detection, and design tailored intervention and prevention strategies to improve the prognosis of psychosis.

## Material And Methods

### Search strategy

The protocol was registered on PROSPERO (CRD42022333886) and PRISMA guidelines were followed^16^. We employed the PsycINFO, PubMed and SCOPUS databases to search for articles examining the relationship between premorbid adjustment and social cognition published from inception to June 2022. In this review, premorbid adjustment includes both domains: social and cognitive premorbid adjustment. We considered that cognitive premorbid adjustment includes academic premorbid performance, measured by a scale, and premorbid IQ, measured by a cognitive test. Social cognition includes general assessment and each of its components: emotion processing, social perception, Theory of Mind and attributional bias.

The search covered the combination of three concepts: our target population (psychotic disorders or people at risk of psychosis), functioning in the premorbid period of psychosis, and social cognition. The search string used in the three databases was: (“social cognition” OR “ToM” OR “Theory of Mind” OR “attributional style” OR “attribution style” OR “emotional recognition” OR “emotion recognition” OR “facial recognition” OR “emotional processing” OR “social perception” OR “emotion perception” OR “affect perception” OR “affect recognition” OR “mentalizing” OR “mentalising” OR “attributions” OR “social knowledge” OR “social cue recognition” OR “emotion identification”) AND (“premorbid adjustment” OR “premorbid functioning” OR “premorbid deterioration” OR “premorbid”) AND (“first-episode schizophrenia” OR “first-episode psychosis” OR “early psychosis” OR “schizophrenia” OR “first episode psychosis” OR “non-affective psychosis” OR “nonaffective psychosis” OR “psychosis” OR “at-risk” OR “ultra-high risk” OR “clinical risk” OR “high risk”). Boolean operators were used to perform a more specific search. Three authors (SO, PP and AB) inspected the references of the included articles.

Further manual searches of the references of the relevant studies and reviews were undertaken by authors.

### Screening and selection criteria

During screening, all authors reviewed titles and abstracts retrieved. Conflicts were reviewed and discussed until consensus. Doubtful articles were included for subsequent full-text review. In the second phase, two of the authors reviewed each article included in the screening phase. Studies were eligible if (i) the sample included patients with psychotic disorder or at high-risk state for psychosis. Given the growing interest in social cognition during psychosis and in its prodromal phase^13,17–22^, the full spectrum of psychotic disorders and high-risk states were included; (ii) social cognition (or any component), and premorbid adjustment were measured. Any method of assessment was permitted, such as medical chart reviews or school reports to inform about cognitive premorbid adjustment; and (iii) the relationship between premorbid adjustment and social cognition or its components were analyzed. This association might not be the main aim of studies. The search was limited to studies published in English or Spanish. Premorbid adjustment or social cognition analyzed as control variables as well as qualitative designs were excluded.

### Data collection process

The authors independently extracted data from all included articles, and discrepancies were resolved via discussion. Data extracted included: (i) first author and year of publication, (ii) aims, (iii) target population and sample size, (iv) assessment methods, (v) outcome variables regarding the aim of this systematic review; (vi) main findings regarding the aim of this systematic review; (vii) general conclusion through a specific question: Is there a relationship between premorbid adjustment and social cognition, and (viii) risk of bias. The risk bias of the articles included in the review was performed by the authors using the National Heart, Lung, and Blood Institute (NHLBI) Study Quality Assessment Tools^23^. Our research group had previously adapted the instrument to better capture the strengths and weaknesses of the studies^24^. The classification of the observational and cross-sectional studies was: “Good” (more than 9 positive answers), “Fair” (between 7 and 9 positive answers), or “Poor” (below 7 positive answers). The classification of the before and after studies was: “Good” (more than 8 positive answers), “Fair” (between 6 and 8 positive answers), or “Poor” (below 6 positive answers). A different cutoff was decided because, in the case of cross-sectional studies, the total number of items assessed was 14, and in the case of before and after studies, it was 12 items. If any information was not applicable or specified, the final risk of bias score was weighted, taking into account only items with yes/no answers.

## Results

### Study selection

**Fig. 1:**
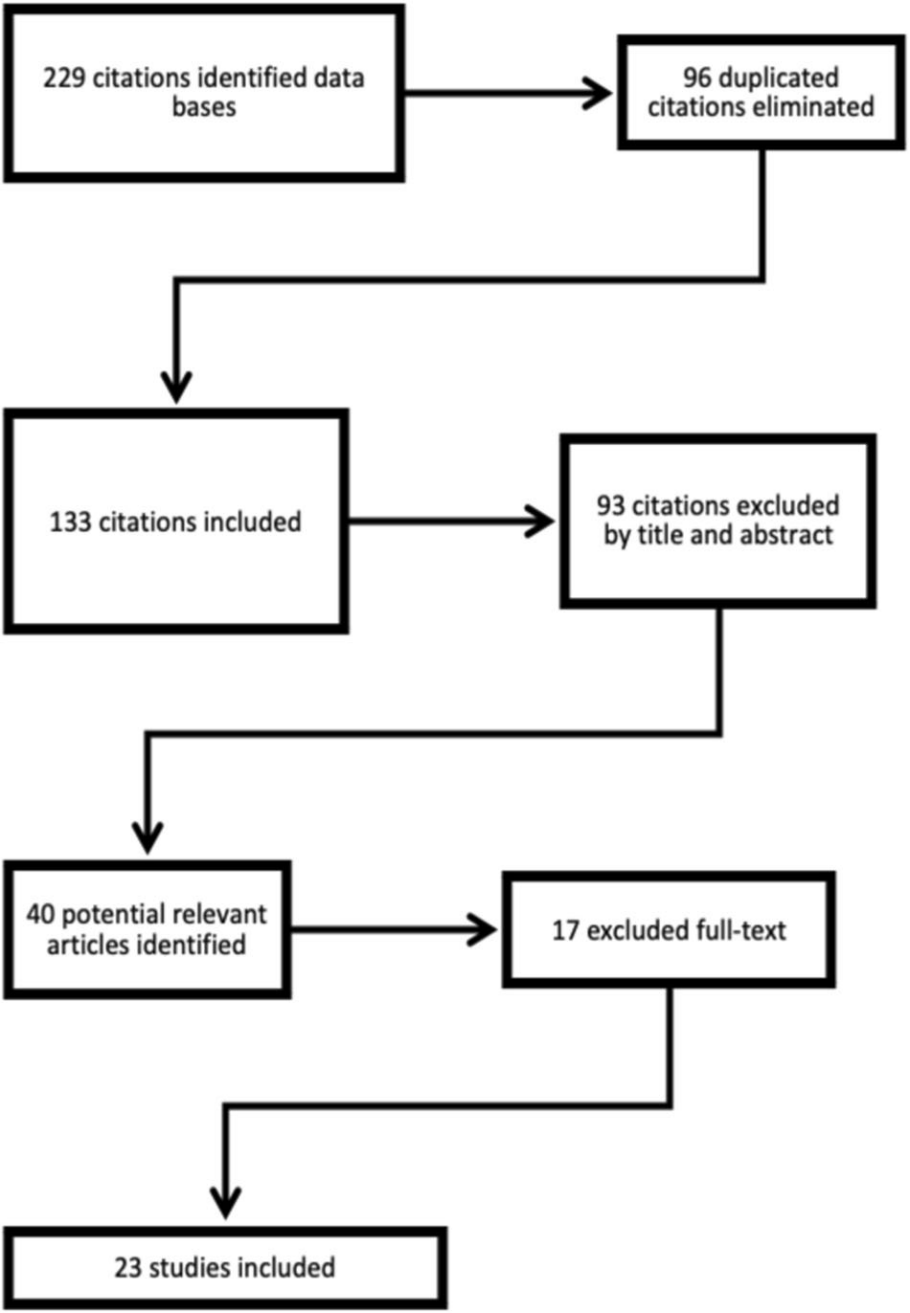
Flowchart for systematic review.

We identified 229 citations. After eliminating duplicates, we included 133 potentially relevant papers. Two evaluators assessed each paper. In a total of 16 papers, two evaluators assessed differently, and the article was reviewed by another member of the team. The percentage of agreement was 88.54% (kappa index of 0.75). After screening, 40 papers were selected, and the full text was retrieved. A total of 23 studies met the inclusion criteria for the final review. Regarding the 17 articles eliminated, 15 of them were because the authors did not analyze the relationship between premorbid adjustment and social cognition, although in 13 of them, they included both constructs, but the aims of the manuscript were different from their relationship. In 2 of them, the sample of the study did not meet the criteria of our review. A flowchart detailing the identification of studies is provided in Fig. 1.

### Study characteristics

Relevant data extracted from the 23 studies are compiled in Table 1. Of them, 14 articles studied premorbid adjustment using the Premorbid Adjustment Scale (PAS)^6^ instrument, and seven assessed premorbid adjustment as premorbid IQ. The remaining articles used other measures, such as medical chart reviews^25^ and the Abbreviated Scale of Premorbid Personal-Social Adjustment^26^. In all the studies, the data indicated premorbid adjustment is obtained based on the previous period of the psychosis onset. In the case of premorbid IQ, it has been estimated using scales that remain stable after the psychosis episode (for instance, vocabulary subtest of WAIS).

**Table 1:** Studies analysing the relationship between social cognition and premorbid adjustment.

Author/year	Aims	Sample	Assessments instruments	Outcome variables	Main findings	Relationship PA and SC (Yes/No)	Risk of bias (Good/Fair /Poor)
Abdel-Hamid et al., 2009	To examine the association of symptom clusters and individual symptoms with ToM performance in schizophrenic patients, in order to test the hypothesis that selectivity of ToM deficits in SCH depends on the predominating symptoms.	Fifty patients diagnosed with SCH (*n* = 43) or schizoaffective disorder (*n* = 7). Twenty-nine healthy controls with no history of psychiatric disorders and no first-degree relatives with psychotic disorders.	Multiple Choice Vocabulary Test (MWT) Zoo Map Test (BADS) WCST Picture Completion Task (HAWIE-R) Computerized ToM test PANSS	Premorbid IQ Executive measures (planning skills; cognitive flexibility; formation to hypotheses) ToM Psychotic symptoms	Significant interaction between ToM total score and premorbid IQ was found (*r* = 0.377; *p* < 0.007). Differential associations of ToM deficits with psychopathology in SCH were found: the disorganization and affective dimensions were associated with ToM deficits.	Yes	Good
Bechi et al., 2019	To identify different premorbid trajectories; to relate them with symptoms, ToM, comparing with controls. To relate with autistic traits.	97 Patients with SCH (*n* = 97); Healthy controls (n = 66).	PAS PST	PA ToM	3 cluster groups: G1: patients with poor PA in childhood, which remained poor in the next stages. G2: patients with a good PA during childhood, which got a further decline across both early and late adolescence. G3: patients with good PA during all stages. Patients in the G1 and G2 were more impaired in ToM than G3 and controls. G3 and controls only differ in total score of PST.	Yes	Good
Bechi et al., 2020	To estimate differences between patients with and without ATs, on demographic and clinical features, cognitive and ToM abilities as well as on daily and premorbid functioning in a sample of patients affected by SCH. To evaluate the efficacy of a ToM training in patients with ATs compared to patients without ATs.	Outpatients with SCH (*n* = 96): ATs+ group *n* = 73 and ATs- group *n* = 23 patients.	PANSS BACS WAIS-R PST PAS QLS	Psychotic symptoms Cognitive abilities ToM PA Quality of life	Differences between groups (ATs+ vs. ATs-) were found for premorbid functioning: a worse functioning during childhood and early adolescence in ATs+ group. ATs+ group manifests lacking cognitive and ToM abilities, associated with the impairment in premorbid and current functioning.	Yes	Fair
Berry et al, 2015	To compare attributional style, ToM abilities and executive skills in paranoid patients and non-clinical controls.	25 acute psychiatric in-patients and 25 healthy nonclinical controls.	IPSAQ Three stories by Frith and Corcoran NART	Premorbid IQ ToM Attributional style	Controlling for the potential Confound of IQ, patients vs controls significant independent predictor of personalizing bias for negative events (β = 0.43, *p* = 0.002), while NART score was not a significant independent predictor (β = 0.24, *p* = 0.069). None in ToM.	No	Good
Bobes et al., 2009	To determine the proportion of ambulatory patients routinely utilizing publicly available mental health care resources in Spain who meet a proposed operational definition of recovery and stability after 1 year. To identify factors that are correlated with meeting such a definition.	1010 patients were recruited in the cross-sectional investigation. Of these, 452 patients were in SR and were followed in the prospective evaluation.	PAS Scale of social cognition for psychosis by GEOPTE	PA SC	Recovery definition is validated by the association of factors: shorter duration of untreated psychosis, better PA and positive attitude/compliance with antipsychotic pharmacotherapy. Additionally, better SC and milder depressive symptoms were associated with an increased chance of recovery.	Not provided	Good
Bridgwater et al., 2022	Explore association between PA and a key component of social cognition (emotion management) during the early phase of schizophrenia.	119 individuals (40 FE-SZ, 22 FE-OP and 57 HC).	PAS MSCEIT	PA SC	For the FE-SZ participants, PAS scores in childhood (ages 5–11) were associated with present-day emotion management on the MSCEIT Managing Emotions scale.	Yes	Good
Bucci et al., 2018	Explore premorbid academic and social functioning in patients with SCH, and its associations with the severity of negative symptoms and neurocognitive impairment.	A group of 915 outpatients with SCH diagnosis, another group of their unaffected first‐degree relatives (*n* = 368; 249 parents, 119 siblings) and one of healthy controls (*n* = 778).	PAS MSCEIT FEIT TASIT	PA SC	All neurocognitive domains, as well as SC index, were negatively correlated with both PAS domains. (Worse PA is associated to worse SC performance). Statistically significant for SC when considering academic PAS domain.	Yes	Good
Buonocore et al., 2018	To identify different clusters of CR in stabilized patients with SCH by measures of IQ and premorbid functioning. To investigate the relationship between CR clusters and clinical, neurocognitive and ToM measures. To analyze the effect of CR clusters on ToM improvement after a socio-cognitive rehabilitation program.	60 patients with chronic SCH.	PAS PST ToM questionnaire	Premorbid functioning ToM	Regarding ToM abilities, results suggested a significant influence of CR. Cluster 3 (high early premorbid functioning trend with within normal limits IQ) showed significantly higher ToM performance, as compared to clusters 1 and 2.	Yes	Good
Casado-Ortega et al., 2021	To compare performance in a measure of SC in people with FEP and HC. To study gender differences. To examine the relation between sociodemographic, clinical and psychosocial variables and SC in the onset of psychosis.	63 people (18 females; 45 males) with a diagnosis of FEP and 78 HC (38 females; 40 males).	Vocabulary Test (WAIS-IV) MSCEIT	Premorbid IQ SC	A positive correlation between premorbid IQ and SC was found.	Yes	Good
Deste et al., 2020	To study the association between ASD symptoms and social cognitive performance.	361 patients with SCH.	WRAT-3 BLERT ER-40 Eye Test TASIT Hinting Task	Premorbid IQ Emotional Recognition ToM	Significant relationship between premorbid IQ and emotional recognition (ER-40 and BLERT) and ToM (Eye Test and TASIT)	Yes	Good
Dewangan et al., 2018	To determine symptom severity and two domains of SC deficit by PA in SCH.	60 males and 60 females with paranoid SCH.	PAS Recognition of facial expression task (Eight standardized photographs) Indian version Picture Arrangement test	PA Emotional recognition Social knowledge	Premorbid poor scholastic performance predictive of poor social knowledge (β = –0.259, *p* = 0.006). Females scored better for recognition of negative emotions (β = 0.194*, p* = 0.029). Premorbid deficit in sociability predicted poor recognition of negative emotions (β = –0.315, *p* = 0.001).	Yes	Fair
Duñó et al., 2008a	To investigate performance on ToM tasks in a group of stabilized SCH patients compared to a control group. To explore the relationship between ToM impairment in 1st and 2nd order verbal experimental tasks with PA measures.	Patients with SCH (*n* = 36) and in-patients into the department of surgery and traumatology (*n* = 31).	PANSS SCL-90 PAS GAF WAIS-III (abbreviated form) Digits backward subtest (WAIS) ToM tasks (first and second order)	PA ToM Psychiatric symptoms Psychotic symptoms Global functioning IQ	Association between poorer PA (social withdrawal) and ToM impairment.	Yes	Good
Duñó et al., 2008b	To evaluate whether first- and second-order ToM deficits in stabilized schizophrenic outpatients were associated with poor PA across different age epochs and several neurocognitive abilities. To establish the relative weight of several variables at predicting ToM deficits.	58 patients with SCH. 48 patients admitted to the Orthopedics and Surgery Department (control group)	PAS Four Theory of Mind false belief tasks: two first-order tasks (The Cigarettes and Sally and Anne), and two second-order tasks (The Burglar and The Ice-Cream Van)	PA ToM	Lower scores on first- (B = −1.990; 95% CI: −3.754; −0.0227; *p* = 0.027) and second- (B = −14.003; 95% CI: −26.340; −1.666; *p* = 0.026) order ToM tasks were associated with a general tendency to show a worse social premorbid functioning. Second-order ToM tasks were more discriminative than first-order ones in relation to poor PA across the different age epochs. Problems in SC and interpersonal functioning may start during infancy in subjects who will develop schizophrenia later on.	Yes	Good
Duñó et al., 2009	To ascertain if ToM deficits in stabilized schizophrenic patients with poor PA, were associated with a history of suicide attempts.	57 schizophrenic patients were recruited and divided in two groups: “suicidal attempts group” (at least one attempt over their lifetime) and “no-suicidal attempts group”.	PAS Four false belief ToM tasks (first-order and second-order tasks)	PA ToM	Mentalizing anomalies significantly predicted suicidal attempt history, but this link vanished when adjusting for PA in childhood and early adolescence.	No	Fair
Gopalakrishnan et al., 2015	To evaluate the factors influencing socio-occupational functioning and well-being in SCH patients following an acute exacerbation of psychotic symptoms.	40 patients were recruited.	Abbreviated Scale of Premorbid Personal-Social Adjustment Tool SOFS	PA Facial affect recognition Socio-occupational functioning	Poor PA was found to be associated with a poorer socio-occupational functioning.	No	Fair
MacBeth et al., 2011	To establish the distribution of secure and insecure attachment representations in FEP. To explore the relationship of attachment and mentalization to symptomatology. To explore the relationship of attachment and mentalization to onset, engagement and adaptation variables in FEP.	34 patients with a FEP.	PAS Level of reflective functioning based on the AAI	PA: social and academic functioning components Reflective functioning	No significant relationships emerged between Reflective Function and mean premorbid social and academic adjustment.	No	Good
Macbeth et al., 2014	To explore correlations between metacognition, symptoms and premorbid functioning in a FEP sample.	20 males and 14 females from Early Intervention for Psychosis FEP sample.	MAS-R PAS	Metacognition PA	Early adolescent social adjustment was significantly associated with poorer Understanding others Mind-subscale of MAS-R (r= −0.402; *p* = 0.03); and nearly significant with understanding own mind (r= −0.359; *p* = 0.051).	Yes	Fair
Marsh et al., 2013	To investigate the feasibility of implementing SoCog-MSRT in a rehabilitation setting and to evaluate whether this training methods produced improvements in SC measures.	17 participants diagnosed with SCH or schizoaffective disorder.	PANSS NART Digits forwards and backwards (WAIS) Recognition of basic facial emotion cues RMET Hinting Task FBPST IPSAQ EQ	Psychotic symptoms Premorbid IQ Working memory Social cognitive measures (recognition of facial emotions; attributional style; ToM; etc.)	Improvements on the IQ was associated with improvements on PST-FB (r = 0.599; *p* = 0.024) and improvements on PST-MC (r = 0.555; *p* = 0.039). Patients with lower IQ may has impact negatively on their ability to benefit from training.	Yes	Good
Piovan et al., 2016	To explore relationship between ToM and social functioning in a group of outpatients suffering from SCH, taking into account variables as figurative language abnormalities, clinical symptomatology and general intelligence.	30 outpatients diagnosed with SCH and 24 healthy subjects were recruited.	TIB (Italian adaptation of NART) PST PSP (Updated version of SOFAS)	Premorbid IQ ToM Social functioning	Regards to general intelligence, a significant correlation with affective but not with cognitive ToM subcomponent arose.	Yes	Good
San et al., 2007	To explore the prevalence of symptomatic remission among patients with SCH, their social-occupational functioning, and the association between these two latter with clinical features such as depressive symptoms and quality of life.	1010 SCH outpatients	PAS GEOPTE scale of SC for psychosis	PA SC	Reported association between a better PA and both symptomatic remission and adequate social-occupational functioning. Functional outcomes are related to the level of social ability.	Not provided	Good
Schenkel et al., 2005	To examine whether deficits in ToM are associated with impairments in context processing, greater disorganized symptoms, poorer premorbid social functioning, and an earlier age of disorder onset.	42 patients (with SCH *n* = 23; with schizoaffective disorder *n* = 19).	Hinting Task Medical chart reviews (a history of academic and social functioning)	ToM Premorbid social functioning	Poorer performance on the Hinting Task was associated with poor premorbid social functioning (t(40) = 3.86; *p* < 0.0001). Childhood social functioning variable made significant contribution to the overall model (*p* < 0.005) in order to predict Hinting Task performance.	Yes	Fair
Trauelsen et al., 2016	To explore association between metacognition and positive and negative symptoms.	101 FEP and 101 controls matched by gender.	MAS-A PAS	Metacognition PA	No relationship between PAS and MAS-A.	No	Good
Uren et al., 2017	To identify cognitive and SC subgroups in FEP comparing to control group, and to relate this cluster to symptoms and functioning.	133 FEP participants and 46 healthy controls.	PST False Belief and Deception Stories Task (Stories) Hinting Task DANVA Faces and Voices subtests WRAT-4	Premorbid IQ SC (ToM, Faces recognition…)	3 clusters: G1: FEP with lower scores in cognition. G2: regular cognition. G3: not impaired cognition. Differences between G1 and G2 were found in the Stories task and the DANVA task. G2 and G3 had differences in almost all domains. The Stories Task was the only significant cognitive measure to distinguish G1 and G2 (*p* = 0 .009). Differences between G3 and G2, were found in the Stories Task (*p* = 0.001). G1 had the poorest IQ performance, followed by G2, and G3 had the highest mean.	Yes	Fair

Note. *AAI* Adult Attachment Interview, *ASD* Autism Spectrum Disorders, *ATs* autistic traits, ATs + with autistic traits, *Ats*- without autistic traits, *BACS* Brief Assessment for Cognition in Schizophrenia, *BADS* Behavioral Assessment of the Dysexecutive Syndrome battery, *BLERT* Bell Lysaker Emotion Recognition Task, *CAARMS* Comprehensive Assessment of At-Risk Mental States, *CNB* Computerized Neurocognitive Battery, *CR* Cognitive Reserve, *DANVA* Diagnostic Analysis of Nonverbal Accuracy-2-Adult Version, *EQ* Empathy Quotient, *ER* Emotional Recognition, *ER-40* Penn Emotion Recognition Test, *FBPST* False-Belief Picture Sequencing Test, *FEIT* Facial Emotion Identification Test, *FEP* First-Episode Psychosis, *FE-SZ* First Episode of Schizophrenia, *FE-OP* First Episode of another Psychotic Disorder, *GAF* Global Assessment of Functioning, *GEOPTE* Grupo Español para la Optimización del Tratamiento de la Esquizofrenia, *HAWIE-R* Hamburg Wechsler Intelligence Test Revised, *HC* Healthy Controls, *IPSAQ* Internal Personal and Situational Attributions Questionnaire, *IQ* Intelligence Quotient, *MAS-A* Metacognition Assessment Scale-Abbreviated, *MAS-R* Metacognition Assessment Scale-Revised, *MSCEIT* Mayer Salovey Caruso Emotional Intelligence Test, *MWT* Mehrfachwahl Wortschatz Test, *NART* National Adult Reading Test, *PREMORBID ADJUSTMENT* Premorbid Adjustment, PANSS Positive and Negative Syndrome Scale, *PAS* Premorbid Adjustment Scale, *PMA* Premorbid Adjustment, *PSP* Personal and Social Performance, *PST* Picture Sequencing Task, *PST-FB* Picture Sequencing Test for False Belief, *PST-MC* Picture Sequencing Test for Mechanical Control, *QLS* Quality of Life Scale, *RMET*= Reading the Mind in the Eyes Test, *SCH* Schizophrenia, *SOCIAL COGNITION* Social Cognition SCL-90 Symptheory of mind Checklist-90, *SoCog-MSRT* Mental-State Reasoning Training for Social Cognitive Impairment, *SR* symptomatic remission, *SOFS* Socio-Occupational Functioning Scale, *TASIT* The Awareness of Social Inference Test, *TIB* Brief Intelligence Test, *ToM* theory of mind eory of Mind, *WAIS-R* Wechsler Adult Intelligence Scale Revised, *WAIS-III* Wechsler Adult Intelligence Scale Third Version, *WCST* Wisconsin Card Sorting Test, *WRAT-3* Wide Range Achievement Test third edition, *WRAT-4* Wide Range Achievement Test fourth edition.

Regarding social cognition, 18 studies analyzed specific domains: 12 studies included Theory of Mind, and six measured more than one domain of social cognition. Five studies used a global measure of social cognition.

As for diagnosis, 17 articles used a sample of individuals with schizophrenia, and six studies included a sample of people with first-episode psychosis (FEP).

In 13 articles, the relationship between premorbid adjustment and social cognition was not the main aim of the study. However, 10 studies explored this question as the main objective^7,25,27–34^.

### General social cognition and premorbid adjustment

A total of five studies considered social cognition as a global construct, using three different assessments: a general scale to measure social cognition in psychosis (GEOPTE)^35^, the Mayer-Salovey Emotional Intelligence Test, MSCEIT^36^, and a social cognition index obtained from the Facial Emotion Identification Test (FEIT)^37^ and The Awareness of Social Inference Test (TASIT)^38^ scores.

In a cross-sectional study on schizophrenia, Bucci et al.^28^ found that individuals with poorer premorbid adjustment (specifically in academic PAS) exhibited lower scores in social cognition (r = −0.19; *p* < 0.0001), as assessed by FEIT (emotional recognition) and the TASIT (Theory of Mind). Additionally, two separate cross-sectional studies analyzed social cognition (using GEOPTE) and premorbid adjustment (using PAS) as predictors of remission in individuals with schizophrenia (San et al.^39^ and Bobes et al.^40^). It should be highlighted that the primary aim of these manuscripts was not to assess the relationship between social cognition and premorbid adjustment. However, they found that both constructs independently contribute to predicting remission.

Two other cross-sectional studies examined social cognition, using the MSCEIT in samples of FEP, founding a relationship between both concepts. Casado-Ortega et al.^33^ found a positive correlation between premorbid IQ and social cognition (r = 0.406; *p* = 0.004) and Bridgwater et al.^34^ described that premorbid maladjustment in childhood was correlated with deficits in emotion processing (r = 0.397; *p* = 0.011).

### Theory of mind and premorbid adjustment

Considering all the subcomponents of social cognition, Theory of Mind is the most highly related to premorbid adjustment, having a total of 13 articles. Most of them, 11 found significant associations between premorbid adjustment and Theory of Mind. One study did not find any association between premorbid IQ and Theory of Mind^41^ in acute in-patients with schizophrenia. Another study found that Theory of Mind and premorbid adjustment explain higher likelihood of suicidality^42^ in patients with schizophrenia, but the authors did not explore the relationship between both concepts directly.

Regarding those articles that found a relationship between premorbid adjustment and Theory of Mind all are based on samples of schizophrenia patients. Duñó et al.^30^ in a cross-sectional study found a tendency to worse performance in first-order tasks of Theory of Mind in early adolescence (χ2 = 5.35; *p* = 0.069) and late adolescence (χ2 = 6.64; p = 0.073) premorbid periods and second-order tasks of Theory of Mind in early adolescence (χ2 = 5.6; *p* = 0.061) and late adolescence (χ2 = 4.69; *p* = 0.096). Furthermore, Duñó et al.^31^ in a cross-sectional study found that lower scores in first-order (B = −1.990; CI: −3.754, −0.0227; *p* = 0.027) and second-order (B = −14.003; CI: −26.340, −1.666; *p* = 0.026) tasks of Theory of Mind were associated with worse social premorbid adjustment from infancy through adolescence. A cross-sectional study^43^ reported that participants with a diagnosis of schizophrenia and autistic traits manifested worse premorbid adjustment than people with schizophrenia without autistic traits, especially during childhood (F = 7.584; *p* = 0.007) and early adolescence (F = 7.338; *p* = 0.008). A previous study by the same authors^27^ based on a cross-sectional study reported that people with schizophrenia with poorer premorbid adjustment presented more impairment in Theory of Mind than those with better premorbid adjustment. Similarly, Buonocore et al.^29^ in a before and after study found that people with schizophrenia with high early premorbid adjustment and IQ within normal limits had significantly better performance in tasks of Theory of Mind than those who had either worse premorbid adjustment and mild intellectual impairments or average/high premorbid adjustment but moderate intellectual impairment (X^2^ = 9.57; *p* = <0.001). In this line, Schenkel et al.^25^ in a cross-sectional study found that poorer performance on Theory of Mind, measured by the Hinting Task, was associated with poor premorbid social functioning (t(40) = 3.86; *p* < 0.0001) in people with schizophrenia.

A significant interaction between Theory of Mind and premorbid IQ was described in five articles. Piovan et al.^44^, in a cross-sectional study, found that in outpatients diagnosed with schizophrenia, premorbid general intelligence significantly correlated with the affective but not with the cognitive Theory of Mind subcomponent (r = 0.553; *p* = 0.002). Abdel-Hamid et al.^45^, in a cross-sectional study, found a significant interaction between Theory of Mind and premorbid IQ (r = 0.377, *p* = 0.007) in people with schizophrenia or schizoaffective disorder. Using a similar sample, Marsh et al.^46^ using a cross-sectional study found similar results, showing that improvement on the Picture Sequencing Test for False Belief (PST-FB) (r = 0.599; *p* = 0.024) and on the Picture Sequencing Test for Mechanical Control (PST-MC) (r = −0.751; *p* = 0.002) were associated with premorbid IQ. In the same line Deste et al.^47^ described that, in people with schizophrenia, a higher premorbid IQ predicted a better mental state attribution (Theory of Mind) performance, as measured by the Reading the Mind in the Eyes Test (Model F = 87.362, R2 = 0.434, *p* < 0.001), and TASIT (Model F = 43.784, R2 = 0.340, *p* < 0.001). Regarding the relationship between Theory of Mind and premorbid IQ in FEP, Uren et al.^48^, in a cross-sectional study, described that this domain is the only one that discriminates between clusters based on premorbid IQ adjustment (*p* = 0.009).

Another important construct related to Theory of Mind is mentalization or metacognition^32^, operationalized as reflective functioning and the ability of the individual to understand and infer mental states of both themselves and others^49^. One cross-sectional study analyzed the associations between social and cognitive premorbid adjustment, and the individuals’ reflective functioning^49^ in people with FEP, but they did not find significant results. Two other cross-sectional studies assessed metacognition with contradictory results^32,50^. One of them did not find significant associations between premorbid adjustment and the subscales of Awareness of the Mind of the Other^50^. However, MacBeth et al.^32^, using a modified version of the Metacognition Assessment Scale (MAS-R)^51^ found a significant association between poor early adolescent premorbid adjustment and poor Understanding of the other’s Mind (r = −00; *p* = 0.03).

### Emotional recognition (or emotional processing) and premorbid adjustment

Four studies assess emotional recognition and premorbid adjustment in samples of people with schizophrenia.

Only two studies directly investigated the relationship between emotion recognition and premorbid adjustment^7^ and premorbid IQ in people with schizophrenia^47^. The first one, Dewangan et al.^7^, in a cross-sectional study, found that poor social premorbid adjustment predicts poor recognition of negative emotions (B = −0.315; *p* < 0.001). The second one, Deste et al.^47^, found that a higher premorbid IQ predicted a better emotion processing performance, as measured by the Penn Emotion Recognition Test (ER-40) (Model F = 27.031, R2 = 0.241, *p* < 0.001), and as measured by the Bell Lysaker Emotion Recognition Task (BLERT) (Model F = 70.269, R2 = 0.381, *p* < 0.001).

Conversely, Marsh et al.^46^, found that, although improvements in Theory of Mind correlated with premorbid IQ, emotion recognition did not improve after a social cognitive training program in a sample of people with schizophrenia or schizoaffective disorder. Similarly, Gopalakrishnan et al.^26^, in a cross-sectional study, reported that in people with schizophrenia, premorbid adjustment but not facial emotion recognition is related to poor socio-occupational functioning.

Other components of Social cognition: Attributional Style and Social Perception or Social Knowledge.

Two studies have analyzed the relationship between premorbid adjustment and attributional bias and social perception/social knowledge. Only the cross-sectional study of Dewangan et al.^7^ analyzed the relationship between social perception/social knowledge with premorbid adjustment in people with paranoid schizophrenia. The authors found that premorbid poor cognitive performance predicts poor social knowledge (β = –0.259, *p* = 0.006). The relationship between premorbid adjustment (using premorbid IQ) and attributional bias was analyzed only in one cross-sectional study^41^, finding no relationship between both constructs in a sample of people with schizophrenia^7^.

### Risk of bias

Regarding the risk of bias, most of the studies included in the review (17 articles) were scored as good. Only 6 studies were classified as “fair”, suggesting some methodological limitations^7,26,32,42,43,48^. Information about the items’ scores is provided in Table 2.

**Table 2:** Risk of bias.

Author/year	Type of research design	Item 1	Item 2	Item 3	Item 4	Item 5	Item 6	Item 7	Item 8	Item 9	Item 10	Item 11	Item 12	Item 13	Item 14	Overall
Abdel-Hamid et al., 2009	Observational and Cross-sectional	Yes	Yes	Not specified	Yes/Yes	No/No	Yes	Yes	Yes	Yes	Not applicable	Yes	No	Not applicable	Yes/Yes/Yes	11.5: Good
Bechi et al., 2019	Observational and Cross-sectional	Yes	Yes	Not specified	Yes/Yes	No/No	Yes	Yes	Yes	Yes	Not applicable	Yes	No	Not applicable	Yes/Yes/Yes	11.5: Good
Bechi et al., 2020	Before and after studies	Yes	Yes	Yes	Yes	Yes/Yes	Yes/Yes	Yes	No	No	Yes	No	Yes	xxx	xxx	9: Fair
Berry et al, 2015	Observational and longitudinal	Yes	Yes	Not specified	Yes	No	Yes	Yes	Yes	Yes	Yes	Yes	Not specified	No	Yes	11.7 Good
Bobes et al., 2009	Observational and Cross-sectional	Yes	Yes	Yes	Yes/Yes	Yes/Yes	Yes	Yes	Yes	Yes	Yes	Yes	Not specified	Yes	Yes/No/Yes	14: Good
Bridgwater et al., 2022	Observational and Cross-sectional	Yes	Yes	Yes	Yes	Yes/Yes	Yes	No	Yes	Yes	No	Yes	No	No	No/Yes/No	10:Good
Bucci et al., 2018	Observational and Cross-sectional	Yes	Yes	Not specified	Yes/Yes	Yes/Yes	No	Yes	Yes	Yes	No	Yes	Not specified	Not applicable	Yes/Yes/Yes	11.5: Good
Buonocore et al., 2018	Before and after studies	Yes	Yes	Yes	Yes	Yes/Yes	Yes/Yes	Yes	Not specified	Yes	Yes	No	Yes	xxx	xxx	10.9: Good
Casado-Ortega et al., 2021	Observational and Cross-sectional	Yes	Yes	Yes	Yes	Yes/Yes	Yes	Yes	Yes	Yes	No	Yes	Not specified	Yes	Yes/Yes/Yes	12.9 Good
Deste et al., 2020	Observational and Cross-sectional	Yes	Yes	Yes	Yes	Yes/Yes	Yes	Yes	Yes	Yes	No	Yes	Not specified	Yes	Yes/Yes/Yes	12.9 Good
Dewangan et al., 2018	Observational and Cross-sectional	Yes	Yes	No	Yes	No	No	Yes	Yes	Yes	Not applicable	Yes	No	Not applicable	Yes/Yes/Yes	9.3 Fair
Duñó et al., 2008a	Observational and Cross-sectional	Yes	Yes	Not specified	Yes/Yes	Yes/Yes	Yes	Yes	Yes	Yes	Not applicable	Yes	No	Not applicable	Yes	12.7: Good
Duñó et al., 2008b	Observational and Cross-sectional	Yes	Yes	Not specified	Yes	No	Yes	Yes	Yes	Yes	Not applicable	Yes	No	Not applicable	Yes/Yes/Yes	11.5 Good
Duñó et al., 2009	Observational and Cross-sectional	Yes	Yes	Not applicable	Yes/Yes	No/No	No	No	Yes	Yes	No	Yes	No	Not applicable	Yes/Yes/Yes	8.2: Fair
Gopalakrishnan 2015	Observational and Cross-sectional	Yes	Yes	No	Yes/Yes	Yes/Yes	No	No	Yes	Yes	No	Yes	No	Not applicable	Yes/Yes/Yes	8.6: Fair
MacBeth et al., 2011	Observational and Cross-sectional	Yes	Yes	Not specified	Yes/Yes	Yes/Yes	Yes	Yes	Yes	Yes	Not applicable	Yes	No	Not applicable	No/No/No	11.5: Good
Macbeth et al., 2014	Observational and Cross-sectional	Yes	Yes	No	Yes	No	No	Yes	Yes	Yes	No	Yes	No	Not applicable	No/No/No	7.5: Fair
Marsh et al., 2013	Observational and Cross-sectional	Yes	Yes	Not specified	Yes/Yes	No/No	Yes	Yes	Yes	Yes	No	Yes	No	Yes	No/No/No	9.7: Good
Piovan et al., 2016	Observational and Cross-sectional	Yes	Yes	Not applicable	Yes/Yes	No/Yes	No	No	Yes	Yes	No	Yes	Not specified	Not applicable	Yes/Yes/Yes	10.2: Good
San et al., 2007	Observational and Cross-sectional	Yes	Yes	Not applicable	Yes/Yes	Yes/Yes	No	Yes	No	Yes	No	Yes	No	Not applicable	Yes/No/Yes	9.3: Good
Schenkel et al., 2005	Observational and Cross-sectional	Yes	Yes	Not specified	Yes/Yes	Yes/Yes	Yes	Yes	No	Yes	Not applicable	Yes	No	Not applicable	No/No/No	10.2: Good
Trauelsen et al., 2016	Observational and Cross-sectional	Yes	Yes	Not specified	Yes	No	Yes	Yes	Yes	Yes	Not applicable	Yes	No	Not applicable	No/No/No	10.2 Good
Uren et al., 2017	Observational and Cross-sectional	Yes	Yes	Yes	Yes	No	No	Yes	Yes	Yes	Not applicable	Yes	No	Not applicable	No/No/No	9.3 Fair

Items of the NIH Quality Assessment Tool for Observational and Cross-Sectional Studies	
Item 1	Was the research question or objective in this paper clearly stated?
Item 2	Was the study population clearly specified and defined?
Item 3	Was the participation rate of eligible persons at least 50%?
Item 4a/4b	Were all the subjects selected or recruited from the same or similar populations (including the same time period)? / Were inclusion and exclusion criteria for being in the study prespecified and applied uniformly to all participants?
Item 5a/5b	Was a sample size justification, power description, or variance and effect estimates provided? / Was sample size ≥ 30 per group?
Item 6	For the analyses in this paper, were the exposure(s) of interest measured prior to the outcome(s) being measured
Item 7	Was the time frame sufficient so that one could reasonably expect to see an association between exposure and outcome if it existed?
Item 8	For exposures that can vary in amount or level, did the study examine different levels of the exposure as related to the outcome (e.g., categories of exposure, or exposure measured as continuous variable)?
Item 9	Were the exposure measures (independent variables) clearly defined, valid, reliable, and implemented consistently across all study participants?
Item 10	Was the exposure(s) assessed more than once over time?
Item 11	Were the outcome measures (dependent variables) clearly defined, valid, reliable, and implemented consistently across all study participants?
Item 12	Were the outcome assessors blinded to the exposure status of participants or not involved in the care of the patient?
Item 13	Was loss to follow-up after baseline 20% or less?
Item 14a/14b/14c	Were key potential confounding variables measured and adjusted statistically for their impact on the relationship between exposure(s) and outcome(s)? / Was a matched control group? / If regression analysis was not possible, were other analyses performed to report potential confounding variables?

Items of the NIH Quality Assessment Tool for Before and After Studies	
Item 1	Was the study question or objective clearly stated?
Item 2	Were eligibility/selection criteria for the study population prespecified and clearly described?
Item 3	Were the participants in the study representative of those who would be eligible for the test/service/intervention in the general or clinical population of interest?
Item 4	Were all eligible participants that met the prespecified entry criteria enrolled?
Item 5a/5b	Was a sample size justification, power description, or variance and effect estimates provided? / Was sample size ≥ 30 per group?
Item 6a/6b	Was the test/service/intervention clearly described and delivered consistently across the study population? / Was a previous similar content intervention controlled for in the analysis if it existed?
Item 7	Were the outcome measures prespecified, clearly defined, valid, reliable, and assessed consistently across all study participants?
Item 8	Were the people assessing the outcomes blinded to the participants’ exposures/interventions?
Item 9a/9b	Was the loss to follow-up after baseline 20% or less? / Were those lost to follow-up accounted for in the analysis?
Item 10a/10b	Did the statistical methods examine changes in outcome measures from before to after the intervention? / Were statistical tests done that provided p values for the pre-to-post changes?
Item 11	Were outcome measures of interest taken multiple times before the intervention and multiple times after the intervention (i.e., did they use an interrupted time-series design)?
Item 12	If the intervention was conducted at a group level (e.g., a whole hospital, a community, etc.) did the statistical analysis take into account the use of individual-level data to determine effects at the group level?

Overall formula: Number of items answered “yes” divided by number of items answered “yes” and “no”, multiplied by number of total items.

## Discussion

We have reviewed literature assessing the relationship between premorbid adjustment and social cognition. A large proportion of the evidence collected in this review shows a significant relationship between these two constructs, specifically between Theory of Mind and premorbid adjustment. In this sense, the lack of an adequate development of cognitive and social premorbid performances, especially in the early stages of the life cycle, has been related to difficulties in processes and functions that allow people to understand the interpersonal world once the onset of psychosis occurs. This evidence involves clinical and research implications. On the one hand, it provides evidence of the need for preventive intervention programs in school stages, focused on improving social and cognitive skills. On the other hand, these results require more research to determine the causality of the relationships found. The results obtained are interpreted below, showing the clinical and research implications derived from each of the components of social cognition analyzed.

Few studies have observed no relationships between social cognition and premorbid adjustment, suggesting that the relationship between these constructs is quite common. In fact, the lowest scores in the risk of bias (scored as “fair”) were obtained in two of the studies that did not find an association between premorbid adjustment and social cognition^26,42^. In the same way, there are two studies^39,40^, that did not provide information regarding a possible relationship between social cognition and premorbid adjustment. Additionally, they evaluate social cognition with a general measure (GEOPTE scale), which does not make the results comparable with studies analyzing social cognition by components. Social cognition is not a unitary construct, including several domains that should be assessed separately.

Considering Theory of Mind and premorbid adjustment, Duñó et al.^31^ found that, in patients with schizophrenia, second-order Theory of Mind tasks were more discriminative than first-order ones in relation to poor premorbid adjustment across different age periods, indicating that second-order Theory of Mind tasks may be more suited to assess mentalistic abilities. Possibly, second-order Theory of Mind tasks are more cognitively demanding, and their deficit may already be indirectly reflected as poor premorbid adjustment in earlier development periods. In fact, Healey et al.^52^ suggested that the decline in Theory of Mind occurs in the reverse order of which the abilities were acquired. In this line, people with lower scores in first and second-order Theory of Mind tasks tended to present worse social premorbid adjustment in infancy and adolescence. This suggests that deficits in Theory of Mind may be trait-like features of people with poor premorbid adjustment who will develop psychosis^18^. This fact can also be supported by studies showing a significant positive interaction between Theory of Mind and premorbid IQ^44–48^. However, further studies will be needed to determine the causality of this relationship. Besides, some of the studies that have reported results in line with relating both constructs present low methodological quality^25,27,48^.

Regarding the relationship between premorbid adjustment and mentalization, a study noted a significant association with poor early adolescent social premorbid adjustment and poor Understanding of the other’s Mind^32^. These metacognitive difficulties may reflect psychodevelopmental factors. As suggested by Peterson & Wellman^53^, early adolescence may be a period of maximum demand in terms of social experiences, and of maximum development of Theory of Mind abilities. However, alternative hypotheses, such as the one held by Tierney & Nelson^54^, suggested an explanation based on “experience-dependent plasticity”: there is no one period that is more sensitive to relevant experience for the development of Theory of Mind, it is feasible at any age, providing the necessary stimulating experiences. In this sense, the possibility of intervening as soon as possible is left open to offer experiences that stimulate Theory of Mind capacity^53^, specifically in individuals with premorbid social and cognitive difficulties. More research is required to expand the evidence and to elucidate the relationship between premorbid adjustment and mentalization, considering that the only study found in this review has methodological limitations.

Emotional processing is another component of social cognition that may already be affected in the premorbid phase of psychotic disorders. The scarce evidence has shown the predictive character of premorbid sociability and cognitive performance in relation to the severity of positive symptoms, social knowledge and deficit in negative emotion recognition in schizophrenia^7^. People with poor premorbid adjustment may have been less exposed to social interactions, diminishing their opportunities to identify and discriminate facial and vocal emotions, and to manage their own emotions. Consequently, a lack of social experience may result in inadequate development of emotion processing, even before the onset of the disorder. A previous study^46^ has shown a lack of effectiveness of psychosocial interventions in emotion recognition in patients with schizophrenia, perhaps due to a deficit in social functioning that should be established beforehand. However, this hypothesis needs more evidence to be confirmed, as well as higher quality studies since some of the limited evidence found presents methodological limitations, such as the study of Dewangan & Singh^7^.

In addition, social knowledge has also been related to premorbid adjustment in patients with schizophrenia, although the evidence is scarce and shows low methodological quality^7^. We hypothesize that social perception/knowledge may be an implicit construct in the academic environment. Consequently, good cognitive performance could facilitate learning social rules and increase opportunities for social experiences. Therefore, it could be feasible to improve areas associated with social perception, such as social skills, community functioning, and social problem-solving^15,55^. Likely, social perception has been less studied because of the limited number of measures, the complexity of the construct, and the difficulty in isolating it from other social cognition domains, such as Theory of Mind^14^.

Frequently, individuals with persecutory delusions attribute negative outcomes to others, rather than to situations. This is known as a personalizing bias^56^. There seems to be no association between personalizing bias and premorbid IQ, as reported by Berry et al.^41^. Although there is an association referred to the morbid phase, between attributional style and cognitive aspects such as IQ^57^ or cognitive flexibility^58^. Nevertheless, data on premorbid adaption and how individuals explain the causes of events are limited to draw solid conclusions. It is described that poor cognitive premorbid adjustment is neurodevelopmentally determined in people with schizophrenia and with stable impairment over time. Therefore, it would be interesting to know whether a poorer cognitive premorbid adjustment is related to a maladjusted attributional style^59^. Furthermore, attributional biases should also be interpreted in relation to social premorbid adjustment. Less social competence during the premorbid phase of the disorder may reflect attributional biases that lead to conflicting social experiences. In contrast to this hypothesis, Garety and Freeman^60^ suggested that attributional style may more closely resemble a thinking style associated with personality rather than a performance-based social cognitive skill.

In this review, various overlapping constructs have been mentioned^49,50^. These refer to the ideas that people form about themselves and others and include social cognition, mentalization, and metacognition. Both constructs, mentalization and metacognition, are confused and could be interchangeable in some studies^61^. However, this review aims to overcome this limitation that some studies have indicated, showing the nuances of each of these constructs: metacognition is a broad concept that refers to the dynamic process of integrating the information obtained through recognition and understanding of one’s own and others’ cognitive processes. Mentalization is closer to metacognition because it is not a static capacity but rather one that changes within inter and intrapersonal contexts^62^. Both concepts refer to the ability to understand one’s own cognitive processes and those of others, and not so much in the content of thought, but the concept of mentalization is restricted to the context of attachment relationships. However, social cognition is a more specific term because it is focused on the accuracy of how social events are interpreted, which is a static assessment^63^. Social cognition encompasses 4 domains: emotion processing; social perception, Theory of Mind and attributional style. We have included these two terms, mentalization and metacognition, when they were related to Theory of Mind. In short, more scientific evidence that contemplates this conception of the terms and allows obtaining results that can be interpreted in an integral way is needed.

Given the importance of premorbid adjustment and social cognition, and the significant relationship between these two variables, it seems that a thorough assessment of premorbid adjustment could be beneficial to tailor personalized interventions and preventive strategies to improve the prognosis of psychosis. Currently, the existing evidence on the efficacy of early intervention programs aimed at people with an incipient psychotic disorder is well-known. These types of interventions include strategies to improve access to mental health care, as well as comprehensive evaluation and intervention strategies that make it possible to adjust to the needs of each patient and thus improve the course of the disorder, even avoiding its appearance.

Early psychological interventions, such as metacognitive training addressed to improve social cognition, should be delivered specially in those people with worse premorbid adjustment. Metacognitive training, which is addressed to increase self-reflection on thought processes and sowing doubts in patients about their non-adaptive beliefs, has shown their effectiveness regarding some components of social cognition such as Theory of Mind and attributional style in patients with recent-onset psychosis^64^. Furthermore, it is possible that deficits in social cognition already appear in the phases prior to the onset of FEP. In this sense, disturbances of social cognition have been considered by some authors as a possible trait marker of schizophrenia^18^. Social cognitive impairments would have trait-like qualities that precede the onset of the disorder and are candidate endophenotypes for schizophrenia^17,19–22^. These impairments could be already present in the premorbid phase and be manifested subtly, in the form of poorer premorbid adjustment. In this sense, social cognition may be considered as a vulnerability marker for psychosis to be contemplated in psychosis detection algorithms to reduce false positive identification. A review article^22^ on social cognition in the ultra-high-risk population concluded that deficits in certain social cognitive abilities appear to be present.

We found some limitations while working on this review, for example, that most of the retrieved literature focused on only two components of social cognition: Theory of Mind and emotion recognition. One of the reasons for it is the availability of assessment instruments. However, it should be noted that there was a great variability among the included articles in instruments to measure both constructs. Likewise, there was considerable diversity in the assessment of premorbid adjustment using different instruments (PAS or medical chart reviews^25^, and Brief Intelligence Test^44^ or NART^41^, among others, respectively), with the PAS^6,65,66^, being the most complete instrument used. Besides, some studies analyzed premorbid adjustment considering social and cognitive domains. Others assess premorbid adjustment only considering premorbid IQ. This variability in the measurement instruments may be behind the lack of consensus in some studies that do not find a relationship between social cognition and premorbid adjustment. Another explanation for the lack of consensus in some studies could be due to the retrospective nature of the premorbid adjustment variable. A high percentage of patients with psychotic disorders have cognitive impairment, including memory difficulties. This can make it difficult to remember information that occurred in previous phases to the onset of the disease. Finally, another limitation is that we did not include the concept of “cognitive reserve” in the search terms, as an umbrella term for premorbid adjustment, but this term was not included because it can refer to cognitive domains before and after the onset of schizophrenia. In the same vein, we have not included in the search MESH terms; however, we have included those relevant words related to our aim.

In the light of the results of this review, we make the following recommendations for future studies: a) The assessment of social cognition and premorbid adjustment should be consensual to reduce heterogeneity factors that may hinder comparability of results across studies. In this sense, regarding social cognition, we recommend assessing specifically the subcomponents rather than the global construct, placing greater emphasis on the study of the domains of social perception and attributional style. With regard to premorbid adjustment, the most widely used scale has been the PAS that allows the assessment of premorbid adjustment across the life cycle and by sub-components. The PAS scale is an objective instrument and measures broader concepts than premorbid IQ, but we recommend not using the general subscale of the PAS for this purpose, as some items assess aspects related to the morbid phase^66^; b) According to a previous study^8^ supporting the idea of considering the premorbid adjustment as a broad concept that encompasses not only social and cognitive aspects, other areas of the person’s life, such as family, vocation, personal well-being, interests in life, energy levels, economic independence and also those related to the way social information is processed, should be taken into account when clinical professionals are exploring the premorbid phase. In this sense, social cognition aspects should also be assessed in the premorbid phase of psychosis, among others; c) Due to the heterogeneity of the assessment methods used in the studies comprising this review, it has not been possible to perform a meta-analysis. In the future, it will be necessary to meta-analyze the influence of premorbid adjustment on the mental processes behind social interactions; and finally, d) In order to improve the quality of the information collected about the premorbid phase, more studies should be carried out focusing on people in a high-risk state of psychosis. In addition, a greater number of prospective and longitudinal studies should be carried out in the future, which would have their starting point in the prodromal phase, making way for causality.

## Conclusions

This comprehensive review reveals a noteworthy connection between certain domains of social cognition, particularly Theory of Mind, and premorbid adjustment. However, there is a need for additional research to explore the relationships involving social perception/knowledge and attributional style. It is crucial to emphasize that longitudinal studies focusing on the early phases of psychosis, before its onset, would provide valuable insights. There is a suggestion to examine functioning in premorbid phases, considering not only social and cognitive functioning but also in terms of social cognition.

The findings indicate that interventions such as metacognitive training should be considered in the individualized therapeutic plans for patients with psychotic disorders who have experienced an impoverished premorbid phase. It is also recommended that future studies address and meta-analyze the influence of premorbid adjustment on social cognition.

### Reporting summary

Further information on research design is available in the Nature Research Reporting Summary linked to this article.

### Supplementary information

Reporting summary
